# Mycotoxins Contamination in Rice: Analytical Methods, Occurrence and Detoxification Strategies

**DOI:** 10.3390/toxins14090647

**Published:** 2022-09-19

**Authors:** Ana Rita Santos, Filipa Carreiró, Andreia Freitas, Sílvia Barros, Carla Brites, Fernando Ramos, Ana Sanches Silva

**Affiliations:** 1 Faculty of Pharmacy, University of Coimbra, Polo III, Azinhaga de Sta Comba, 3000-548 Coimbra, Portugal; 2 National Institute for Agricultural and Veterinary Research (INIAV), I.P., Av. da República, 2780-157 Oeiras, Portugal; 3 Associated Laboratory for Green Chemistry of the Network of Chemistry and Technology, REQUIMTE/LAQV, R. D. Manuel II, Apartado 55142, 4051-401 Porto, Portugal; 4 GREEN-IT Bioresources for Sustainability, ITQB NOVA, Av. da República, 2780-157 Oeiras, Portugal; 5 Centre for Animal Science Studies (CECA), ICETA, University of Porto, 4501-401 Porto, Portugal; 6 Associate Laboratory for Animal and Veterinary Sciences (AL4AnimalS), 1300-477 Lisbon, Portugal

**Keywords:** co-occurrence, HPLC-MS, mitigation, mycotoxins, QuEChERS, rice

## Abstract

The prevalence of mycotoxins in the environment is associated with potential crop contamination, which results in an unavoidable increase in human exposure. Rice, being the second most consumed cereal worldwide, constitutes an important source of potential contamination by mycotoxins. Due to the increasing number of notifications reported, and the occurrence of mycotoxins at levels above the legislated limits, this work intends to compile the most relevant studies and review the main methods used in the detection and quantification of these compounds in rice. The aflatoxins and ochratoxin A are the predominant mycotoxins detected in rice grain and these data reveal the importance of adopting safety storage practices that prevent the growth of producing fungi from the Aspergillus genus along all the rice chain. Immunoaffinity columns (IAC) and QuECHERS are the preferred methods for extraction and purification and HPLC-MS/MS is preferred for quantification purposes. Further investigation is still required to establish the real exposition of these contaminants, as well as the consequences and possible synergistic effects due to the co-occurrence of mycotoxins and also for emergent and masked mycotoxins.

## 1. Introduction

Mycotoxins are secondary products resulting from toxigenic fungal metabolism. They consist of low molecular weight metabolites and are mostly produced by the genus *Aspergillus*, *Fusarium,* and *Penicillium* [1]. Over 400 types of mycotoxins have been identified, but attention is mainly given to those with the greatest public health relevance, such as aflatoxins (AFs), ochratoxin A (OTA), fumonisins (FUMs), trichothecenes (TCs) and zearalenone (ZEA) [1,2]. Their structural diversity results in different chemical and physicochemical properties, and they are associated with the development of acute and chronic problems such as carcinogenicity, teratogenicity, mutagenicity, and hepatotoxicity [1,3].

Due to their worldwide prevalence and their association with health disorders, mycotoxins have been recognized as a major health and economic issue [4]. In fact, these toxins are considered by European Food Safety Authority (EFSA) as a threat and are one of the most reported hazards on RASFF (Rapid Alert System for Food and Feed) [5].

The European Commission (EC) has established a regulation where the maximum levels allowed for some mycotoxins are established, but many studies have reported cases where those limits are exceeded [6]. Therefore, and due to climate change, strict control is required, as well as the development and validation of suitable analytical methods [7].

It is almost impossible to avoid the presence of mycotoxins in the food chain, but their levels can be controlled by the implementation of good agriculture practices and decontamination processes [1].

The present review comprises a review of the most commonly found mycotoxins in rice and the main methods used for their extraction, detection, and quantification, as well as the techniques used in decontamination processes. 

## 2. Mycotoxins

Over 400 mycotoxins have been identified to date, but only a few represent known concerns to human health, including AFs, OTA, DON, T-2/HT-2 toxins, FUMs, and ZEA [1].

Aflatoxins are a family of mycotoxins produced by a fungus of the genus *Aspergillus* (mainly *A. flavus* and *A. parasiticus*), which can be found in rice [2]. Among all classes of mycotoxins, aflatoxins are thought to be the most toxic, and the greatest concern, not only at economic level (mainly in the United States and European Union) but also in health terms, contributing to hundreds of hepatocellular carcinoma (HCC) cases every year in developing countries [8,9]. 

The most relevant aflatoxins are aflatoxin B1 (AFB1), aflatoxin B2 (AFB2), aflatoxin G1 (AFG1), aflatoxin G2 (AFG2), and aflatoxin M1 (AFM1), with aflatoxin B1 being the most commonly occurring and toxic one [10]. 

Ochratoxins are produced by *Aspergillus* or *Penicillium*, mainly *A. ochraceus* and *P. verrucosum*, under variable environmental conditions. Ochratoxin A is known to be the most toxic and prevalent in this class [10,11]. Ochratoxins are found to be stable in acidic conditions and elevated temperatures. This thermal resistance makes them difficult to eliminate under normal cooking conditions [11]. OTA is considerably prevalent in cereals [12].

Fumonisins are fungal toxins produced by *Fusarium* spp. (mainly *F.verticilloides* and *F. proliferatum*), found most frequently in maize and cereals. This class of mycotoxins is known to be non-fluorescent and hydrophilic, unlike other classes, that can be completely dissolved in organic solvents [11]. There are more than 28 known fumonisins, divided into four main groups: A, B, C, and P. The fumonisins B group is the most frequent in nature and comprises fumonisin B1 (FB1), fumonisin B2 (FB2), and fumonisin B3 (FB3), with FB1 being the most toxic and frequent member of the family (70–80% of all fumonisins) [13].

TCs are a group of mycotoxins mainly produced by fungal species of *Fusarium* spp. This family is organized into four groups: the trichothecenes A, B, C, and D, each with structurally related toxins. Types A and B TCs are the most frequent in the group [14]. Type A TCs are the most toxic and include T-2 and HT-2 toxins. These toxins are mainly produced by *F. lansehtiae*, *F. sporotrichioides, F. poae,* and *F. acumminatum*, and have been detected in many food matrices including barley, oat, wheat, rice, and maize [13]. Type B TCs include nivalenol and deoxynivalenol (DON), with the last one being the most frequent, although less toxic, of the group. DON is predominantly produced by *F. culmorum* and *F. graminearum* and can be found in cereal and cereal-based products, widely distributed [14]. Of all classes of mycotoxins, trichothecenes are the most structurally diversified, and mainly contaminate cereals, such as maize, rice, oats, wheat, and barley [15].

Zearalenone (ZEA) is a macrocyclic lactone produced by multiple species of *Fusarium*, mainly *F. graminearum*, *F. sporotrichioides* and *F. semitectum.* It is usually associated with maize crops, but it can also be found in other cereals such as wheat, barley, rice, and oats. This toxin tends to appear mostly in temperate and warm countries with high humidity levels [13]. ZEA’s contamination usually occurs concurrently with DON or, less frequently, with aflatoxins. This mycotoxin can be partially eliminated under elevated temperatures but is stable under normal cooking conditions [16]. 

### 2.1. Emerging Mycotoxins

Emerging mycotoxins can be defined as a group of mycotoxins that has not been routinely determined or legislatively regulated, but the evidence of their incidence has been rapidly increasing in the last few decades [16].

Enniatins (ENNs) and beauvericin (BEA) are structurally related mycotoxins that belong to this class, produced by many filamentous fungi. ENNs are mainly produced by *Fusarium* spp., *Alternaria* spp., *Halosapheia* spp., and *Verticillum* spp., while BEA is mostly produced by *Beauveria* spp., *Paecilomyces* spp., *Polyporus* spp., and *Fusarium* spp. [17,18]. These emerging mycotoxins have been reported in several matrices in recent publications, but their toxic effects have not yet been well established. The main source of contamination of these mycotoxins are cereals (including maize, wheat, barley, and rice), not only for being ideal matrices for fungal growth but also because of their great consumption among the population [17].

To date, 29 enniatin analogs have been reported, with enniatin A (ENN A), A_1_ (ENN A_1_), B (ENN B), B_1_ (ENN B_1_), and B_4_ (ENN B_4_) being the most prevalent, but there have also been found lower amounts of enniatins C, D, E, and F. Their structural differences are responsible for the distinct bioactivities of these analogs [18].

Studies have shown that emerging mycotoxins are prevalent worldwide and are able to co-occur with other classes of mycotoxins. Therefore, they might be a hazard to human and animal health. There have been no reports found on mycotoxicosis caused by BEA and ENNs, although some studies have described possible risks associated with their ingestion due to their ionophoric properties. Further investigation needs to be done in order to evaluate their health risk and eventually come up with regulatory levels [17,18]. 

Moniliformin (MON) and sterigmatocystin (STC) are also emerging mycotoxins that have also already been reported in rice. STC has the particularity of being a precursor of AFB1, and so they share a similar mechanism of toxicity, by forming Deoxyribonucleic acid (DNA) adducts and generating reactive oxygen species (ROS). This can lead to false negatives or underdetermination of AFB1 since STC can be later converted into its successor, considered by many authors as the most toxic and concerning mycotoxin [16]. *Alternaria* toxins, such as alternariol and tenuazonic acid, and citrinin (CIT) are other examples of emerging mycotoxins, mostly detected in fruits and vegetables [16].

### 2.2. Masked Mycotoxins

Masked mycotoxins are produced by plant enzymes involved in detoxification processes or during food processing through conjugation with polar substances such as glucose, sulfate, and amino acids. This structure modification leads to difficulty in their detection by conventional analytical methods [17,19].

Deoxynivalenol-3-glucoside (DON-3G) and ZEA-14-glucoside (ZEA-14G) are among the most commonly detected conjugates. Those conjugations are an attempt of the plants to make the compounds more soluble in water for faster elimination, and they usually exhibit lower toxicity in comparison with parent forms [20].

When metabolized, the masked mycotoxins suffer hydrolyzation and release the original mycotoxin. This can also happen during processing and constitutes a concern, because masked mycotoxins are not being accounted for by analytical methods and a food commodity that was judged as compliant might become non-compliant at a later stage, because of the release of the mycotoxin [19].

### 2.3. Co-Occurrence

Co-occurrence consists of the occurrence of multiple mycotoxins within the same food matrix [2]. Multiple exposures are very frequent, being even more common than the presence of a single mycotoxin [10]. Although there is still a lot to know, the co-occurrence of mycotoxins may result in additive or synergistic effects, increasing the toxicity of the contaminated material [2].

In rice, the occurrence of different mycotoxins and their metabolites is unavoidable due to the simultaneous infection with multiple fungi, that are toxigenic, i.e. they are able to produce multiple mycotoxins [21].

AFB1 and AFB2 are the most frequently documented as co-occurring mycotoxins, but it has also been reported that the co-occurrence of mycotoxins is produced by different fungi species [21]. It has been described in several studies the combined effects of mycotoxins; however, it is still unknown the nature of the observed effects, the relative potencies of each mycotoxin, and the way those interactions could enhance their respective toxic effects [10,17].

### 2.4. Mycotoxins-Producing Fungi

Mycotoxin-producing fungi mostly belonging to the genera *Aspergillus*, *Fusarium* and *Penicillium* are among the organisms able to contaminate rice [15].

Fungi growing conditions are dependent on many factors, such as the presence of fungal inoculum on susceptible crops, fertilization balance, insect damage, inadequate storage conditions, temperature, humidity, water activity (a_w_), pH and nutritional composition of the food product, and so their relevance is different around the world [21,22]. Weather variables are the leading factors contributing to mycotoxin occurrence, but the cropping system used is a powerful tool for farmers to mitigate grain contamination [23].

Even inside the same genera, different species may grow during different stages of production [15].

*Aspergillus* grows predominately in tropical countries, with high temperatures paired with high values of RH and a_w_. For example, rice in tropical Asia is mostly contaminated with *Aspergillus* fungi (such as *A. flavus* and *A. ochraceus*) because of the conditions during pre-harvest (improved crop management and agronomic practices, control of insects that favor fungal infection, host plant resistance, and biological control, such Afla-Guard^®^ GR from Syngenta^®^ (Iowa, United States) that can be used in maize, which active ingredient is a nontoxigenic strain of *A. flavus* that acts by competitively displacing toxigenic, aflatoxin-producing strains, something similar should be specific to rice), harvest and postharvest stages [21,22]. Despite being difficult to predict the occurrence of fungal diseases and toxin contamination in food grains predictive models can be used and most publications on predictive mycology have just come up during the last decade [22,24,25]. A model is a simplified representation of a system, which is a limited part of reality and contains interrelated elements and attempts to summarize the main processes, put forward hypotheses, and verify their coherence and consequences [22]. Prediction models have been developed, based on several impact factors that might influence mycotoxins occurrence. Especially, in terms of the effect that climate change may have nowadays in the future, those models have been used to calculate the associated risks for human and animal health and with these models, the final levels of mold or mycotoxins contamination may be predicted (a useful tool for the food industry) [24,25].

*Fusarium* spp. grows under high temperatures and moisture and is the major cause of a decline in rice quality during cultivation due to environmental conditions [15]. *Penicillium* spp. is not found in the field during the growing period, and their contamination is usually associated with rice storage conditions [15].

It is well known that not all fungi are threatening and not all their secondary metabolites are toxic. Mycotoxins’ toxicity depends not only on their producer but also on their interaction with each other and with other microorganisms, on the edaphoclimatic conditions, and on the system of farm management (organic versus conventional) to which they are submitted [21]. Moreover, fungal contamination of certain food matrices, is not a synonym of contamination with mycotoxins, since fungi only produce these metabolites under specific circumstances as a strategic defensive mechanism. Therefore, the production of mycotoxins might not be associated with the presence of the fungal itself, but with the presence of other fungi or microbes, or even with the fluctuation of the environmental conditions (such as water availability and temperature) [26].

### 2.5. Factors Associated with Rice Contamination by Mycotoxins

Food contamination by mycotoxins is dependent on the presence of fungi, the application of unsuitable agricultural practices and the conditions of harvesting, and storage. Since most mycotoxins are thermostable and consequently able to persist under food processing and cooking temperatures, the key to their absence must be based on the prevention of their occurrence [27].

Mycotoxins’ contamination may occur in different stages, from pre-harvest to post-harvest steps, during processing, packaging, distribution, or even storage. The rice grain is harvested with husk and their physical structure exerts a protective effect against field mycotoxin contaminations. Usually, mycotoxins’ contamination in rice grain is associated with fungal growth due to improper storage conditions [28].

Despite the protective grain layers, paddy rice is susceptible to contamination after harvesting, since almost of worldwide rice production is harvested in subtropical environments (under warm and humid conditions), and then stored for large amounts of time before its consumption. When stored under inappropriate conditions it constitutes a great substrate for fungal growth. According to the Food and Agriculture Organization (FAO), every year around 15% of the rice harvest is lost due to fungal growth and mycotoxin contamination [29,30].

Rice crop development is strongly dependent on temperature since it has a great impact on plant photosynthesis, which when submitted to temperature stress, suffers a reduction in physiological activity. Therefore, climate change may have a substantial impact on rice grain production. Along with temperature increase, projections point to a decrease in precipitation along the Mediterranean basin area, which should have a negative impact on this crop, since it is very dependent on water supply [31,32].

Climate changes are also increasing mycotoxins’ contamination. Earth temperature is expected to increase 1.5 to 4.5 ºC until the end of the 21st century. Global warming boosts water evaporation from the surface, which results in an increase in moisture within the atmosphere. Consequently, an increase in the fungal population and mycotoxins’ occurrence is expected since temperature and humidity are key factors for their growth [32].

### 2.6. Toxicity and Mechanisms of Action of Mycotoxins

Mycotoxins’ contamination is associated with multiple risks to human health due to their toxicity, in particular their carcinogenicity. In order to avoid these risks, taking into account epidemiological, experimental, and mechanism studies, the International Agency for Research on Cancer (IARC) has come up with a scale of hazard assessment of mycotoxins in human health [33].

Mycotoxin ingestion can result in both acute and chronic toxicity. Acute toxicity is associated with a rapid toxic response, while chronic toxicity is a result of low-dose exposure over a long period. Although chronic toxicosis has been found to be a global problem, acute toxicosis is more common in developing countries, particularly in Africa [8,33].

Aflatoxins have carcinogenic, mutagenic, hepatotoxic, teratogenic, and immunosuppressive effects, with the liver being the most affected organ. AFB1 is the most toxic of all aflatoxins, with AFB2, AFG1, and AFG2 having, respectively, 50, 20, and 10% of its toxigenic power [17]. Aflatoxins have been classified by IARC as a Group 1 carcinogen, due to the high risk of development of HCC after chronic exposure. AFM1 is a result of AFB1’s biotransformation and has been classified as a Group 2B (possibly carcinogenic to humans). In humans, acute aflatoxicosis usually results in abdominal pain, vomiting, pulmonary and cerebral edema, coma, convulsions, or even death [33,34]. 

After being ingested, aflatoxins are biotransformed in the liver by a family of enzymes called CYPP450. These are responsible for turning AFB1 into its carcinogenic form: AFB-8,9-epoxide. This metabolite is able to form adducts with cellular macromolecules, such as DNA, which results in a modification of its structure and biological activity, and therefore in the carcinogenic and mutagenic effects of the toxin. A mutation of gene p53 seems to be the base of the association between aflatoxins and HCC, and this type of cancer is found to be more prevalent in regions with high consumption of aflatoxins [35].

In countries with a high rate of hepatitis B virus (HBV), exposure to AFB1 may constitute an even bigger issue, since the risk of liver cancer development after exposure to aflatoxins in HBV-positive people is about 30 times greater than in HBV-negative people [27].

Ochratoxin A is a fat-soluble mycotoxin that has been classified by IARC as Group 2B (possible human carcinogen) and is associated with immunotoxicity, neurotoxicity, genotoxicity, and embryotoxicity in both humans and animals [34,36]. Its toxicity seems to be related to its structural similarity with phenylalanine, an essential amino acid. OTA inhibits proper protein synthesis in the kidney and liver, by interfering with phenylalanine hydroxylase. It also seems to interfere with DNA and RNA synthesis [36].

Fumonisins are classified by IARC as belonging to Group 2B (possibly carcinogenic to humans) and seem to be associated with esophageal tumors and liver toxicity [34,37]. FB1 is found to be the most abundant and toxic of the group, followed by FB2 and FB3. Recent studies have been focusing on FUM’s mechanism of action, and their similarity to sphinganine and sphingosine has come to attention with their possible role in the inhibition of sphingolipids biosynthesis. These sphingolipids are allocated on the membrane of eukaryotic cells and are responsible for the formation of secondary messengers, involved in the regulation of several cellular processes such as gene expression and protein activation/deactivation. By disrupting these mechanisms, this class of mycotoxins might contribute to many effects at a cellular level such as apoptosis induction and carcinogenic effects [38]. 

Some studies have correlated the levels of FBs in food with the development of esophageal cancer in humans. Moreover, they also seem to be associated with brain and spinal cord neural tube defects, when ingested at high levels during pregnancy [38].

ZEA is frequently described as an estrogenic mycotoxin due to its structural similarity to estrogens. Because of that, ZEA and its metabolites are able to bind competitively to estrogen receptors, activate the estrogen gene, and induce reproductive disorders. Long-time exposure to ZEA has also been shown to be associated with liver lesions and HCC development in the worst cases [39]. ZEA is associated with cytotoxic, hematologic, genotoxic, hepatotoxic, and immunotoxic effects, and has been classified by IARC as a group 3 carcinogenic (not classified as human carcinogenic) due to reduced evidence in experimental animals and inadequate evidence in humans [4,34].

Trichothecenes can easily penetrate cell membranes and react with cellular organelles and nucleic acids, which justifies their high toxicity. The major mechanism described consists of the inhibition of ribosomal protein synthesis, followed by disruption of DNA and RNA synthesis [40]. 

DON has been found to be immunosuppressant and genotoxic, but due to a lack of evidence of carcinogenicity, was classified by IARC as group 3 carcinogenic (not classified as human carcinogenic) [4,34,37]. Nausea, vomiting, diarrhea, dizziness, and fever are some of the reported effects of human exposure to DON-contaminated grains [41].

T-2 toxins have also been classified as group 3 by IARC, and along with HT-2 toxins, have been associated with a reduction in body weight, liver and kidney toxicity, immunotoxicity, neurotoxicity, and haematotoxic effects [4,34].

### 2.7. Mycotoxins Legislation with Special Focus at EU Level

Due to the global toxic effects of mycotoxins, a vast number of governmental authorities, including the Food and Drug Administration (FDA), World Health Organization (WHO), EFSA, and FAO, are paying attention and setting maximum levels for mycotoxins in foodstuffs, in order to protect human health [9]. The availability of toxicological information and dietary exposure, along with the distribution of mycotoxins and the available analytical methods, are among the factors that influence the regulated levels [42].

In Europe, the maximum levels of mycotoxins are established for the most known and frequently detected ones in Section 2 of the Commission Regulation (EC) No. 1881/2006 of 19 December 2006 and its amendments that sets maximum levels for certain contaminants in foodstuffs. Those limits were fixed according to mycotoxins’ prevalence and toxicity, and are established for several molecules, such as AFs, OTA, DON, ZEA, and FMs in many food matrices [6]. This regulation was amended in 2010 by the Commission Regulation (EU) No. 165/2010 of 26 February 2010 which established new AFs maximum levels in foodstuffs. Before the milling process, the levels are expected to be slightly higher, due to the greater fraction of mycotoxins in bran, that are removed during this process, lowering the concentrations to an acceptable level [21,43]. The levels established for cereals by the Commission Regulation (EC) No. 1881/2006 of 19 December are described in Table 1.

As a result of the protective layer of husk in paddy rice, low levels of *Fusarium* toxins were detected, and this cereal does not have a specific maximum limit as maize. No maximum levels of *Fusarium* toxins (ZEA, FUMs, T-2, and HT-2 toxin) are established for rice but rice is predominant in baby foods for infants and young children formulations that have a specific maximum limit [6]. Due to the harmful effects related to the presence of T-2 and HT-2 toxins in feed and foodstuff, the EC came out with a recommendation (“Commission Recommendation of 27 March 2013 on the presence of T-2 and HT-2 toxin in cereals and cereal products”) where are established the tolerable daily intake (TDI) for some food matrices. Rice and rice products are not included in those matrices because these toxins occur at very low levels in this matrix, and so it was excluded from this recommendation since it does not seem to constitute a health concern [44].

The European Regulation concerning the maximum limits of mycotoxins in foods is more restricted than the rest of the world. Outside the European Union (EU), levels of mycotoxins are regulated according to different legally binding documents, or have no limits at all, depending on the type of mycotoxin and foodstuff. All these limits were described in “Worldwide regulations for mycotoxins in food and feed in 2003 by FAO (2004) [45].

China and India, the main rice producers in the world, have established maximum levels, although those are much higher than those of the EU. China sets a maximum of 10 µg/kg to AFB1 (No limit on the sum of AFs in rice) and in baby food is only 0.5 µg/kg. [46]. In India, the limits for AFs are set at 30 µg/kg, which constitutes a matter of concern to the consumers’ health [46]. Still, other countries, such as the USA, Canada, and Japan, do not have maximum limits for all mycotoxins mentioned above (Table 1). For example, the USA only has limits for the sum of Afs, 20 ug/kg and for the DON, 1000 µg/kg, as well as Japan, but in Japan, the sum of AFs is 10 µg/kg [47,48,49,50,51]. In Canada they have the maximum limits in the order of nanograms, for example, DON has a maximum limit of 2 ng/kg [52].

One of the greatest limitations in the regulations is associated with the fact that the maximum limits are set according to the mycotoxins’ individual toxicity, not taking into account their co-occurrence and potential synergism. 

Due to the high susceptibility of maize to contaminations with *Fusarium*-produced mycotoxins (DON, ZEA, fumonisins) the European Regulation specifies maximum levels for feed and food unprocessed maize (Table 1). The rest of the cereals for direct human consumption, especially rice, have been regulated with more restricted levels in particular if used in baby foods for infants and young children. The knowledge of the occurrence of regulated mycotoxins in rice assumes great importance since rice production is mostly for direct human consumption and simultaneously is highly used in the formulations of baby foods for infants and young children to fulfill their ‘gluten-free’ claims. In addition, a great number of studies have reported rice contamination by several unregulated mycotoxins, so the establishment of maximum limits for more mycotoxins in specific foods seems to be required. 

## 3. Analytical Methodologies to Determine Mycotoxins

Since their first discovery, many methods have been developed for the analysis of mycotoxins in food, despite the frequent analytical challenges. These challenges include difficulties associated with low-level contamination, complex matrices where contamination occurs, evolving complex extraction procedures, the structural diversity of mycotoxins as well as their co-occurrence. In order to face these challenges, many analytical methods have been developed, although they require continuous improvements in order to support mycotoxin legislation and protect human health and the food and feed industry [4].

Mycotoxin determination in food samples is usually associated with common steps, that include sampling, homogenization, sample preparation (extraction generally followed by clean-up), and lastly detection and quantification [4].

### 3.1. Sampling

Sampling is considered a key step in mycotoxins analysis since it is fundamental to ensure the accuracy of the results and to decide if the whole food batch is compliant or not [4,53].

Mycotoxins are not distributed homogeneously in food; therefore, the implementation of a rigorous sampling protocol is of great importance, to guarantee that the analyzed sample is representative of the entire bulk. Considering consumer safety and producer protection, many sampling plans have been established [53]. These plans are instituted by regulatory entities, such as the FDA and the EC, that came up with the Commission Regulation No. 401/ 2006 where the sampling and analysis methods (such as the number and amount of samples) for the official control of mycotoxins in foodstuffs are described [54,55].

Processed products usually require simpler sampling procedures, since mycotoxins are less heterogeneously distributed in these products than in raw agricultural commodities [15].

### 3.2. Extraction and Clean-Up Procedures

Extraction is a step required before most detection and quantification analytical methods [37]. This step is of great importance and consists of the separation of the analytes of interest from the food matrix, frequently followed by a clean-up phase to eliminate possible interferences. In the case of solid food samples, such as rice, the first step consists of the extraction of compounds of interest into a liquid phase, followed by a clean-up step in order to enhance the specificity and sensitivity of the detection method [3].

The mycotoxins’ chemical properties, the nature of the food matrix, and the final method for detection that will be used are three main factors that should be considered in the selection of the methods for extraction and clean-up [4].

The most frequently used extraction technique consists of the extraction using organic solvents: liquid-liquid extraction (in case of a liquid sample) and solid-liquid extraction (in case of solid samples) [55]. Solid-liquid extraction (SLE) is commonly used for mycotoxins extraction from grains and cereals, such as rice. The solvent selection must rely on the polarity of the mycotoxins of interest and on the type of matrix. Mycotoxins are usually soluble in organic solvents (such as chloroform, acetone, methanol, and acetonitrile), but barely soluble in water. Fumonisins are an exception and present high-water solubility. A mixture of organic solvents with water or acidic solvent is commonly used since water enhances the penetration of the organic solvents in the food matrix and the acidic solvent has the ability to break the strong bonds between the analyte and protein and sugar present on the food matrix [13,56]. This method is associated with high recoveries; however, the use of large amounts of sample and organic solvents, as well as the need to use time-consuming purification processes to minimize interferences during the determination, are significant limitations [57]. 

Recent studies have been using solvent extraction methods, such as supercritical fluid extraction (SFE), microwave-assisted extraction (MAE), and accelerated solvent extraction (ASE). In comparison with SLE, these methods are faster, require smaller volumes of chemical solvents, and are associated with better extraction efficiencies, although they might be costly. Before further clean-up steps, sample filtration and centrifugation are required to eliminate possible interfering particles [56].

The clean-up step plays an important role, allowing the elimination of the substances that may interfere with the detection of mycotoxins, and consequently improving accuracy and precision. Some clean-up methods have been described, including solid phase extraction (SPE), immunoaffinity columns (IAC), solid-phase microextraction (SPME), matrix solid-phase dispersion, and the quick, easy, cheap, effective, reliable, and safe (QuEChERS) method [55].

SPE consists of extracting mycotoxins dissolved in an extract (mobile phase) and passing through solid support (stationary phase), where the mycotoxins are absorbed, and some matrix components are eluted. Usually a washing step, before elution, can eliminate some other interferents that might also be adsorbed in the stationary phase. In the final step, elution of mycotoxins is achieved with an organic solvent for which they have a stronger chemical affinity. The solid phase selection depends on the polarity of mycotoxins and the type of matrix [20,56]. This technique is described as safe, efficient, and reproducible, although it has some limitations, such as the fact that the sample has to be in a liquid phase, the low selectivity due to matrix effects, and the impossibility of using the same solid support for all mycotoxins [20].

Immunoaffinity columns are composed of activated solid phase support, bound to a given antibody. When the sample extract passes through the column, mycotoxins bind selectively to the column antibodies, while interferents and other matrix components are removed by a subsequent washing step. After that, the mycotoxin is eluted with a miscible solvent, such as methanol, removing them from the column [58]. This method has great selectivity, although it also presents some disadvantages, such as the high cost, the column being limited to single use, and its ability to only isolate a given type of mycotoxins, or a group of structurally related mycotoxins. Beyond that, there is also the risk of antibody denaturation, while in contact with some organic solvents, or the possibility of cross-reactivity and establishment of non-specific interactions [17,58]. IAC are available for the extraction of the most common mycotoxins such as AFs, ZEA, OTA, FUMs, and DON, and some columns allow the simultaneous extraction of different classes of mycotoxins [58]. For more complex samples, sometimes it is required the combination of IAC with other extraction methods like SPE [59].

The sample preparation method QuEChERS has been used for extraction and clean-up of different food matrices prior to the detection of mycotoxins. This technique includes two different phases: an extraction step (solvent extraction) followed by a purification one (dispersive-SPE) [60]. The first step is based on solvent extraction, using acetonitrile in the presence of salts such as magnesium sulfate (MgSO_4_) and sodium chloride (NaCl), in order to remove water from the organic phase and reduce the number of polar interferences, respectively [56]. For the second phase, a primary/secondary amine (PSA), or C18, is frequently used to retain co-extracted compounds such as lipids, sugars, organic acids, or even some pigments. As described in the name itself, this is a fast, simple, and inexpensive method, that uses small amounts of solvent compared with other methods [56]. 

A compilation of studies that reported mycotoxin’s occurrence around the world is presented in Table 2, along with the respective extraction and purification methods. The most frequently used methods for the extraction step in the compiled studies were QuEChERS, immunoaffinity columns, and SPE, but in the last few years, there has been a growing preference for the QuEChERS method.

OTA contamination was found in levels higher than those permitted in cereals, in multiple studies [2,61,67]. Aflatoxin levels were also found to be above the permitted limits, according to some studies [2,27,62,70]. 

By exploring Table 2, we are once again threatened with the prevalence and unavoidability of mycotoxins’ contamination, since more than one study reported the contamination with at least one mycotoxin in over 80% of the analyzed samples [62,65]. Moreover, methods that have shown to be efficient in removing fungal from foodstuffs, might not be efficient in removing mycotoxins, since Ruadrew et al. found that 1/3 of the analyzed samples were contaminated with aflatoxins, in the absence of Aspergillus [28].

The sample with the greatest mycotoxins levels found in this literature review was reported by Suarez-Bonet et al. in a sample of rice from Spain [63]. The maximum levels of AFB1 and total aflatoxins were respectively 91.7 and 138.6 µg/kg, which far exceed the regulated limits, and the fact that those samples were cultivated in temperate climate region (Mediterranean, Spain) enhances the fact that this is a worldwide problem [63]. The highest contamination with OTA was reported by Manizan et al. in a sample of 15 µg/kg [2]. Furthermore, Manizan also emphasized the co-occurrence of mycotoxins, by finding 8 different mycotoxins in two rice samples [2].

### 3.3. Analytical Methods

#### 3.3.1. Immunochemical Methods

The immunoassay technology has proven to offer many advantages in mycotoxins determination, through the development of simple, efficient, and sensitive methods, based on antibody-antigen reactions. Among these methods are included enzyme-linked immunosorbent assay (ELISA), flow injection immunoassay (FIIA), lateral flow immunoassay (LFIA), flow immunoassay, and chemiluminescence (CL) [72]. 

CL has already been applied in the determination of mycotoxins in maize samples and consists of the production of fluorescence as a result of a chemical reaction [73]. The most reported advantages are the use of simple instrumentation and the low detection limits obtained [74,75].

ELISA is probably the most frequently used of all published immunological-based methods for mycotoxins determination. ELISA kits are available for the detection and quantification of all major mycotoxins and provide rapid screening results, without the need for clean-up and concentration steps, which makes possible its use in field conditions [58].

This technique is based on the interaction between mycotoxins and antibodies marked with toxin-enzyme conjugate for multiple binding sites. The level of color developed is dependent on the amount of antibody-bound toxin-enzyme conjugates. There are two types of ELISA tests: direct and indirect. Direct ELISA provides quick results and, because it uses only one antibody, it reduces cross-reactivity reactions. However, the direct method is associated with less sensitivity, due to the difficulty of signal amplification on the primary antibody. Indirect ELISA recurs to labeled secondary antibodies, providing higher sensitivity, due to signal amplification [71]. This method is specific, rapid, and easy to use, although it has some disadvantages, including the possibility of cross-reactivity occurrence and dependence on a specific matrix (since matrix effect or interference may induce under or overestimation of mycotoxins) and contamination level [15]. Moreover, each kit is designed for a single use and detects only one mycotoxin. In addition, it can become costly when there is the need to identify various mycotoxins and perform multiple tests. High-performance liquid chromatography (HPLC) analysis is often used as a confirmation method after ELISA e CL [58].

#### 3.3.2. Chromatographic Techniques

Chromatographic methods are the most frequently used for mycotoxins analysis in food samples [53].

Thin layer chromatography (TLC) is commonly used as a rapid screening technique in the analysis of some mycotoxins. Thus, recent investigation has been focusing on the application of methods that allow the detection and quantification of multiple mycotoxins with high selectivity and sensitivity, and the achievement of more accurate results [20].

In order to accomplish that, many other techniques have been developed like HPLC coupled with mass spectrometry (MS), fluorescence (FLD), diode array (DAD), or ultraviolet (UV) detectors. Moreover, gas chromatography (GC) coupled with MS, flame ionization (FID), or electron capture (ECD) detectors have been applied in the identification and quantification of volatile mycotoxins like TC. GC is rarely used in the analysis of mycotoxins with low volatility and high polarity since it requires a prior derivatization step [71,76].

Liquid chromatography (LC) is able to separate thermolabile, non-volatile, and substances with different polarities. Moreover, it can differentiate substances with structural similarities, without the need for derivatization steps, that are required in GC [77]. The solid phases placed inside the analytical column in LC can be classified as normal or reverse phases. LC in the normal phase consists of the elution of mycotoxins through a solid phase (composed of a free or covalent-bounded particle of phenyl, aluminum, or silica resulting in a polar stationary phase), using a low polarity solvent like acetonitrile. LC methods for aflatoxin determination include both normal and reverse-phase separations, although current methods for aflatoxin analysis typically rely upon reverse-phase HPLC [78]. In the case of RP-HPLC- Fl, a derivatization step is done in order to increase fluorescence intensity. This step can be a precolumn derivatization with trifluoroacetic acid or a postcolumn derivatization with iodine or bromine [68,79]. The reverse phase consists of hydrocarbonated non-polar solid phases (C_8_, C_18_, or short chain of phenyl, cyanopropyl, and n-alkyl bound to silica surface), through which mycotoxins are eluted using binary polar mixtures of water and organic solvents [57]. In Table 3, a summary of liquid chromatography-relevant detection/quantification analytical methods to determine mycotoxins in rice and rice products is presented. HPLC, coupled with an MS detector, was initially applied to the analysis of single mycotoxins, but to date, it is possible to simultaneously quantify many mycotoxins belonging to various chemical families in a single run, which makes it the method of choice for detecting multiple mycotoxins. The simultaneous detection of multiple mycotoxins is particularly desirable because of the co-occurrence of multiple mycotoxins in food. These modern chromatographic methods may also reach sub-ppb levels of the limit of detection when used following suitable preparation and purification steps [68].

Liquid chromatography, mass spectrometry, and fluorescence were the most used techniques. Although HPLC-FLD is preferred for single mycotoxin determination, HPLC-MS/MS is the preferred method for simultaneous determination of multiple mycotoxins, and according to the studies compilation in Table 3, through the years there is a tendency to employ this method. 

New technologies are being applied for mycotoxin determination, such as Orbitrap and Time-of-Flight (ToF) detectors. These new technologies allow the obtainment of more accurate results, and specifically, quadrupole-Orbitrap has the ability to confirm the presence of a certain compound by its exact mass and to identify metabolites or compounds that have not yet been monitored [83]. Quadrupole-ToF detectors are also being used in mycotoxin determination since they provide exact mass information and determine the presence of unknown compounds in real samples [84]. 

The ultra-high performance liquid chromatography/time-of-flight mass spectrometry (UHPLC/TOFMS) method was developed and validated to screen for the presence of mycotoxins in cereal matrices from Ecuador [84]. Paddy rice was contaminated with AFG1, AFB1, DON, FB1, and polished rice was contaminated with AFG1 and HT-2 toxins, as we can see in Table 3. Since no mycotoxin regulations are enforced in Ecuador, the obtained LODs and LOQs were compared with the European maximum permitted limits (Regulation No. 2006/1881/EC) [82]. 

Fumonisin mycotoxins which are hazardous to humans and animals were produced in a *Fusarium* verticillioides-infected solid rice culture. To decrease the possibility of the formation of artifacts, the fumonisins were analyzed by reversed-phase high-performance liquid chromatography/electrospray ionization time-of-flight (RP-HPLC/ESI-TOFMS) and ion trap mass spectrometry (RP-HPLC/ESIITMS) immediately after the extraction of the culture material, without any further sample clean-up and, this is essential for the separation, detection, and characterization of unknown, structurally related secondary metabolites such as the mycotoxin isomers. These results can serve as a starting point for more detailed examinations regarding the structure, toxicity, and biosynthesis of FB1 isomers, with a view to providing additional knowledge concerning food and feed safety [81].

The methods used seem to be suitable since both limits of detection (LODs) and limits of quantification (LOQs) are below the maximum limits set by the EU. Moreover, we can observe that through the years, LOD and LOQ levels are becoming lower, which is associated with the evolution of the used techniques, which are becoming more sensitive. By the analysis of Table 3, we can also conclude that the lower LOD and LOQ levels were obtained when using liquid chromatography coupled to triple quadrupole MS, which is the current method of election for mycotoxins’ determination in food.

Internal standards are chemical compounds that present a similar behavior to the target substance, and that are not present in the sample, but intend to minimize process losses (like extraction losses). Internal standards are not frequently used in these studies, and only two of the studies did use these standards in their works [13,68]. Because of their chemical and chromatographic similarities to the target toxins, sulfamethoxazole and tagged stable isotopes were chosen as internal standards [13,68].

Regarding the most frequently used detectors in LC, UV detectors have been losing popularity, due to the lack of selectivity and sensitivity, since many interferences absorb in this zone of the spectrum, along with mycotoxins. Diode array detector (DAD), although allows a complete spectrum of all wavelengths, is associated with low sensitivity levels. For mycotoxins that present natural fluorescence (some aflatoxins and OTA), or for those that are fluorescent after derivatization, fluorescence detectors are also a good option since they present high sensitivity and selectivity levels. In spite of those benefits, FLD is being replaced by MS [55].

HPLC coupled with mass-spectrometry has allowed great advances in mycotoxins’ analysis since it offers higher sensitivity and selectivity in comparison with other methods, as well as structural information of the analyzed mycotoxin metabolites or degradation products. That is why an increasing number of researchers have been using this technique, not only for identification and quantification but also for toxicokinetic and metabolism studies [85,86]. The mass spectrometer ionizes the molecules and identifies them based on their mass-to-charge ratio (*m/z*). Based on the ionization technique, different interfaces have been applied in the detection of mycotoxins, such as atmospheric pressure chemical ionization (APCI), atmospheric pressure photo-ionization (APPI), and electrospray ionization (ESI) [87]. Moreover, there are multiple types of mass analyzers, such as triple quadrupole (QqQ), ToF, and ion trap. Each mass spectrometer presents advantages and disadvantages, and its selection is dependent on the purpose of the analysis. QqQ is mainly used in routine analysis, due to its selectivity, robustness, and repeatability, although it is not able to determine unknown compounds. For that purpose, there are other developed instruments such as ToF detectors (which provide exact mass through high-resolution mass spectrometry) or ion trap detectors (which offer a fragmentation schedule, allowing unambiguous identification of the compound) [17]. Triple quadrupole (ESI) is the most commonly used in mycotoxin analysis. ToF and Orbitrap analyzers are becoming more popular due to their high resolution and high accuracy but ToF is more frequently used [4]. In Table 4 we can see a comparison of MS/MS systems, such as TQ MS (Tripe Quadrupole MS), Q-TOF MS (Quadrupole Time-of-Flight MS), and Orbitrap MS.

### 3.4. Biosensors 

Since the first article was published in the biosensor area in 1962, great efforts have been made to their commercialization and use in medicine, pharmacy, agriculture, the food industry, and environmental monitoring [90].

In general, biosensors contain biological or biologically derived sensing elements to detect specific bio-analytes integrated with a transducer in order to convert biological signals into electrical signals [91].

Biosensors with point-of-care features are a promising tool for mycotoxins detection, and many researchers focused on developing disposable biosensors [92].

Related to physicochemical properties of mycotoxins (e.g., fluorescence) or the type of transduction, three groups of biosensors are mostly used: electrochemical (potentiometric, amperometric, and impedimetric), optical (surface plasmon resonance (SPR), and fluorescence) and piezoelectric (quartz crystal microbalance (QCM)). Most of the importance of biosensors relies on their high sensitivity and specificity with minimum sample treatment. Electrochemical biosensors are predominant among the above groups [24,29,89,91]. 

Furthermore, to improve biosensors’ sensitivity, a wide variety of metal nanoparticles, carbon nanotubes (CNTs), nanofibers, and quantum dots (QDs) are used due to their simplicity, physiochemical malleability, and high surface areas [87,92]. 

The electrochemical biosensors are based on potentiometric, amperometric, and impedimetric detection methodologies. The potentiometric sensor requires two (working and reference) or three (working, reference, and counter) electrode systems, and the recognition event is provided by the changes in the circuit potential between working and reference electrodes. The amperometric sensor, similarly to the potentiometric requires a two or three-electrode system [91]. Electrochemical biosensors are a serious alternative to more complex official instrumental techniques such as HPLC coupled to FLD or MS detectors and provide additional benefits allowing reduced costs and shortening analysis time [24,29].

Optical biosensors provide a powerful and attractive alternative to conventional analytical methods such as ELISA and chromatographic techniques which are widely used for the detection of mycotoxins [90]. Optical biosensors can employ numerous optical methods to detect an analyte of interest [90]. Those methods are usually based on light absorbance, fluorescence, light polarization, and rotation or vibration spectroscopy measurements, such as SPR and fluorescence, approaches like fluorescence resonance energy transfer (FRET) [86,87]. The SPR system utilizes a thin metal (silver or gold) film between two transparent media with different refractive indices, such as glass prism and sample solution. The SPR method detects alterations in the surface layer refractive index in contact with the sensor chip. In the FRET system, the energy is transferred from an excited donor fluorophore to nearby acceptor species. The acceptor and donor in the FRET can be designed in biunique or one-to-multiple manners, ensuring the simultaneous application of multiple mycotoxin detection [91].

The QCM transducer consists of thin gold-plated crystal quartz, where electrodes are placed. Molecular recognition and a binding event in the electrode surface lead to mass alteration and specific vibrations when an electric signal is sent by the quartz, which results in inducing alterations in the resonant frequency [91].

The development of universal biosensing systems and multiplex assays is another trend in the development of mycotoxin biosensors. Although it can be achieved in many cases by replacement of bioreceptor, the number of appropriate multianalyte biosensors is very limited [90]. The results are obtained relatively quickly, as the samples do not need to be shipped and analyzed at laboratories. It also prevents slowing down the food production process. The main limitations of these methods are matrix interference, antibody cross-reactivity, and the necessity of matrices’ validation [91]. 

## 4. Mycotoxin Contamination in Rice 

In the EU, the RASFF allows a quick and simple share of information, between food safety entities and the EC members, about food and feed hazards, such as contamination by mycotoxins, pesticide residues or other contaminants, pathogenic microorganisms, or heavy metals [4]. Every time contamination by mycotoxins or other food hazards is found, the RASFF member state that discovered it releases a market notification [92]. RASFF notifications can be provided by different entities, such as non-official market controls, industrial companies controls, border controls, and consumers, or they might even be reported by countries outside the EU [93].

RASFF is a valuable tool, not only because it allows the identification of emerging food safety risks, but it is also possible to check the most frequent occurrences in a certain period [92].

According to RASFF, mycotoxins are the basis of a great number of notifications, being one of the main cited hazards during the last decade. In 2019, 553 notifications were emitted referring to mycotoxins in foodstuffs, and around 84.6% corresponded to AFs contamination [92,94].

Table 5 summarizes the reported notifications related to mycotoxins contamination in raw rice grain (brown, white) and rice flours since 2019. According to this table, since 2019 over 86 occurrences classified as a serious risk were reported, which means the contamination levels exceed the legislated levels, and so they were removed from the market. The highest AFB1 levels reported in this period were found in a batch imported from Pakistan to the Netherlands, where 44 μg/kg was reported for AFB1 and 49 μg/kg for total AFs. These values far exceed the levels regulated by the EC for these mycotoxins in cereals for direct human consumption (2 μg/kg for AFB1, and 4 μg/kg for AFs) [95].

All these findings emphasize the presence and relevance of mycotoxins in food safety discussion and the need for rigorous control for their mitigation in the rice value chain. Moreover, looking at the results we can conclude that there is a higher incidence of notifications in basmati and organic rice. This raises questions: are more risks of rice mycotoxin contamination associated with their origin or organic production?? Additionally, most of the contamination samples were original from countries outside the EU, which emphasizes the need for stricter control of food products coming from foreign countries.

## 5. Contamination Mitigation

Since mycotoxin-producing fungi may affect rice in multiple stages, many strategies to overcome this problem have been developed, from prevention of their occurrence to decontamination methods [96].

One of the developed strategies to reduce mycotoxigenic fungi in the field is chemical control. Although chemicals have shown to be successful in crop protection, they are associated with undesirable effects. By acidifying the soil, they may interfere with the plant’s growth, as they decrease the occurrence of beneficial organisms. Furthermore, nowadays there is an increasing pressure to reduce the use of insecticides, fungicides, and herbicides, in order to achieve higher agricultural sustainability levels [21].

Postharvest strategies are associated with the application of proper storage conditions because almost all mycotoxin contamination in rice grain is associated with inadequate storage. Therefore, the application of suitable packaging practices (such as the use of ultra-hermetic airtight containers), temperature and humidity control, and ventilation efficiency are essential to avoid fungal growth and mycotoxins accumulation [90]. However, brown rice has more nutritional value which motivates the search for other detoxification strategies.

The distribution and concentration of mycotoxins, as well as their physical and chemical properties, suffer modifications during processing, which may lead to a variation in their toxicity levels [17]. Therefore, it is of great importance to understand the impact and phases where those variations occur. Some studies have found higher levels of AFB1 and AFB2 in brown rice and bran, and lower levels in white rice, suggesting the most relevant step to overcome this mycotoxin is bran removal [95].

Since in some cases mycotoxin occurrence cannot be avoided, some decontamination methods have been developed. These methods must be safe, environmentally friendly, effective, and have a good cost-benefit relationship. A decontamination strategy, to be considered effective, must be able to inactivate, remove or destroy the mycotoxins, and retain the nutritional properties of the foodstuff. Moreover, it must not alter the product’s technological properties, or form other toxic substances or metabolites [95].

In the case of aflatoxins, several detoxification strategies have been proposed, such as physical methods of separation, thermal inactivation, irradiation, adsorption from solution, solvent extraction, microbial inactivation, and fermentation, as well as chemical detoxification methods [97].

In summary, three types of decontamination methods may be applied: physical, chemical, or biological. However, there is no single technique that has proved effective against the wide array of mycotoxins that might occur simultaneously in a food commodity. The methods should be able to completely destroy, inactivate, or remove the toxin along with any residual fungal spores. At the same time, it must preserve the nutritional value and the technological properties of the commodity. In short, in Table 6 we have the advantages and disadvantages of the different methods [95,97,98].

Physical methods comprise the separation of damaged or contaminated crops from healthy ones and they include methods such as sorting, sieve cleaning, density segregation, washing, dehulling, and steeping that help reduce the concentration of mycotoxins. They also include the destruction of mycotoxins through heat treatment and irradiation. The study of Reduction in Aflatoxin Content of Feed and Food [99] shows that the removal method of external grain parts (dehulling, polishing) was effective in reducing 88–92% of aflatoxins, high moisture thermal treatment (roasting, extrusion, cooking, high-pressure cooking, instant catapult steam explosion) was effective in reducing 25–88% of aflatoxins in rice and UV-light, near-infrared radiation reduced <99 % of aflatoxins in rice. Although physical techniques seem acceptable since there would be limited change afterward in the properties of the rice grains, their usage is still considered unpractical and limited only to large-scale industries since they might be time-consuming and expensive [95,98].

Other alternatives are chemical methods that employ chemical compound treatments with acids, alkalis, and reducing and oxidizing agents, that are either of organic or synthetic nature. Chemical treatment has shown to be effective in the removal of some mycotoxins, however, some chemicals may not show enough effectiveness in the removal of high levels of mycotoxins. These methods include the use of chlorination agents, oxidants, or hydrolytic agents, and also the use of biological agents such as plant extracts and essential oils (EOs) [100]. 

Treatment with ozone was shown to be promising since it can degrade mycotoxins through reacting with bonds in the mycotoxin chemical structure especially double bonds in mycotoxins such in AFB1 [98].

Although quite a few synthetic preservatives have been identified, their continuous use has been associated with some disadvantages, such as health and environmental issues, an increase in fungal resistance, and allergic reactions. Therefore, the tendency to use natural compounds, such as EOs, to preserve foodstuffs has been increasing in the last decades and is gaining cumulative interest because of their traditional use in pharmaceutics [98,99]. EOs have shown to exhibit biological antifungal, antibacterial, and antioxidant properties, and have already been applied in a wide range of industries, including the pharmaceutical, agricultural, and food ones [101]. Some studies have been performed in order to establish EOs effects on mycotoxigenic fungi and mycotoxin synthesis, and the results indicated that thyme and oregano EOs have been commonly used against fungi producers of aflatoxins, *A. flavus* and *A. Parasiticus* [101,102]. Moreover, cinnamon and cinnamaldehyde have been revealed to present antifungal activity against *Aspergillus* and *Fusarium* genera, and significant antimycotoxigenic activity against DON, AFB1, ZEA, and OTA. Great results using oregano extracts have also been reported against OTA [103,104,105]. Regardless of all these advantages, EOs also present some issues, such as the occurrence of undesirable organoleptic effects and their low potency. In an attempt to overcome their undesirable organoleptic effects, research studies have developed new approaches such as encapsulation and coating. Their low potency is being overcome through their association with other antimicrobial compounds, to obtain synergistic effects [106].

Both physical and chemical methods present disadvantages, since complete decontamination is not achieved, and these methods are associated with high costs and nutritional loss [102].

Lastly, another strategy developed to reduce mycotoxigenic fungi contamination, comprises the use of microorganisms. This biocontrol method is based on multiple mechanisms, including their ability to compete with pathogens for space and nutrients, produce antimicrobial compounds, induce host resistance to the disease, or directly antagonize the pathogen. Lactic acid bacteria have been used as biocontrol agents since they seem to have a great potential to control fungal diseases. A couple of strains of *Streptomyces corchorusii* and *Burkholderia gladioli* have also been studied because of their abilities to produce cell wall degrading enzymes and to inhibit *A. flavus* growth, respectively [21].

Some of these methods have already been applied to rice in order to mitigate mycotoxin contamination, through the application of field and postharvest good practices. Rice processing also constitutes an important step and seems to reduce mycotoxin content, although it cannot fully eliminate these contaminants [21]. EOs have also already been applied in rice, in order to manage mycotoxin formation and fungal growth, and seem to constitute an effective technique. One of the studies was performed by Wan et al. (2019), in order to evaluate the effects of thyme, lemongrass, cinnamon, peppermint, and clove EOs in the production of DON in contaminated rice. These samples were incubated for 5 days in the presence of the previously referred EOs and, by the end of that period, the results indicated several reductions in mycotoxin production [102].

Another study reports chemically characterized Myristica fragrans essential oil (MFEO) as a plant-based food preservative against fungal and aflatoxin B1 (AFB1) contamination of scented rice varieties. Myristica fragrans Houtt. (family: Myristicaceae) is an aromatic plant indigenous to Indonesia, and also cultivated on large scale in India. In this paper, the authors see the efficacy of MFEO against isolated fungal species and AFB1 secretion by AF LHP R14 cells, the antioxidant activity of MFEO, and the phytotoxicity assay of MFEO. Additionally, it shows that due to the pronounced antifungal, antiaflatoxigenic, antioxidant activity, and nonphytotoxic nature, the MFEO can be recommended as a plant-based food preservative for the protection of scented rice varieties and other agri-food commodities from fungal and mycotoxin contamination as well as oxidative biodeterioration [107].

In addition, another paper shows that *Apium graveolens* essential oil (AGEO) and their major components linalyl acetate(LA) and geranyl acetate GA (1:1:1) can inhibit the growth of a wide range of toxigenic food-borne molds as well as AFB1 secretion and recommends its possible deployment for development of novel plant-based safe food preservative [108].

## 6. Conclusions and Future Perspectives

Mycotoxins and their fungal producers constitute a great public health issue, with AFB1 being in the spotlight of those concerns since it was considered by IARC as a group 1 carcinogen. Since the prediction of mycotoxins contamination is very dependent on climate change, the key to minimizing their occurrence must be based on prevention and control. To do so, the implementation of good agricultural and production practices, along with the adoption of proper process, transport, and storage conditions with control analysis of critical points is fundamental. Although agricultural practices and control methods are in constant evolution, a large number of RASFF notifications are still reported every year due to mycotoxins contamination, with some of the values being far above the legislated levels. 

Since 2019, the reported notifications of mycotoxins contamination in rice (86 occurrences) and other published results highlight the aflatoxins and OTA levels as a serious risk and a main concern for the rice chain sustainability. 

To minimize the exposure to mycotoxins, more sensitive and accurate analytical methods for their determination have been developed. IAC and QuECHERS are the preferred methods for extraction and purification and HPLC-MS/MS is the preferred method for quantification purposes. Considering the continuous evolution of methods, it is expected that these techniques will be replaced by high-resolution mass spectrometers such as Orbitrap and ToF. These detectors are still very expensive, but there is a possibility that in the future they will be less expensive and become progressively more ubiquitous in routine laboratories. The development of screening methods with greater precision and sensibility able to be employed in the field is also expected.

Further investigation is still required in this field in order to better understand the effects of mycotoxin co-occurrence and its potential synergism. Moreover, climate changes have been found to be problematic in this research area, since higher temperature and humidity levels are favorable conditions for fungal growth and mycotoxin production. Therefore, it would be of great importance to carry out more studies in order to evaluate the impact of climate change on rice contamination by mycotoxins.

The legislation itself also requires updating since it establishes the maximum levels for mycotoxins in cereals for direct human consumption, but emergent and masked mycotoxins are not considered. 

Rice is one of the most consumed cereals worldwide not only for direct consumption but also for processing into baby foods, resulting in a large exposure to their potential contaminants, consequently, the continuous control of rice mycotoxins occurrence is relevant for their mitigation and avoiding the associated risk to human health.

## Figures and Tables

**Table 1:** Adapted from Commission Regulation (EC) No. 1881/2006 and its amendments, establishing the maximum permitted levels of mycotoxins in cereals [6].

Mycotoxins	Maize Unprocessed (µg/kg)	Cereals for Direct Human Consumption (µg/kg)	Baby Foods for Infants and Young Children (µg/kg)	Ref.
AFB1	5	2	0.1	[6]
Sum of AFB1, B2, G1 and G2	10	4	-	[6]
OTA	5	3	0.5	[6]
DON	1750	750 *	200	[6]
ZEA	200	200	20	[6]
T-2 and HT-2 toxin	200 (indicative TDI level)	100	15	[44]
Fumonisins	2000	1000 **	200	[6]

TDI—Tolerable Daily Intake. * for bread the value is 500 μg/kg; ** for breakfast cereals the value is 800 μg/kg.

**Table 2:** Extraction procedures to determine mycotoxins in rice and rice products and levels of contamination of rice samples.

Type of Sample	Mycotoxins Analyzed	Extraction Method	Extraction Conditions	Number of Samples	Sampling Period	Levels of Contamination (μg/kg)	Conclusions of the Study	Ref.
Organic Rice	OTA	Extraction with MSPD	Sample was blended with the solid phase C8 (2.5 g/1.5 g) until achieving a homogeneous mixture. The mixture was eluted through a column (100 mm × 9 mm i.d. glass column with a coarse frit) using MeOH: FA (99:1, *v*/*v*). The eluate was concentrated using a N_2_ steam, filtered and then centrifuged.	9	April 2005–November 2005	Mean: 2.57 ± 3.43 Range: 2.10–7.60	OTA was present in 4 out of the 9 samples.	[61]
Rice	AFs	SPE	Solvent: ACN: H2O: acetic acid (79:20:1 *v*/*v*/*v*). The supernatant was centrifuged, and a purification step was conducted, diluting the final extract with ACN:water:acetic acid (20:79:1). After a second purification by filtration, the final sample was injected into the UHPLC-MS/MS.	40	January–March 2010	0.15–4.42 (10/40 samples)	80% of the cereal samples were contaminated with at least one mycotoxin; 4% of the samples exceeded the EU regulatory levels for AFs and OTA (4 and 5 μg/kg respectively)	[62]
	OTA					0.2–4.34 (6/40 samples)		
	ZEA					1.5–51.1 (5/40 samples)		
	DON					6.15–34.92 (8/40 samples)		
	FB1					12.59–33.25 (3/40 samples)		
	FB2					12.36–31.19 (3/40 samples)		
	T2					5.88–55.35 (3/40 samples)		
	HT-2					48.18 (1 sample)		
Jasmine Rice	AFs	Immunoaffinity columns	Sample extract: MeOH:H20 (60:40 *v*/*v*) and NaCl. The sample was diluted in distilled water and filtered. IAC: The column was buffered with PBS at a flow rate of 5 mL/min. The sample was then eluted using MeOH and distilled water, at a flow rate of 2 mL/min, and collected in an amber glass vial.	-	-	Mean: 11.4 of total aflatoxins (in the absence of *Aspergillus*)	1/3 of the analyzed samples exceeded the levels of AFs tolerated in the EU.	[28]
Rice	AFB1	Immunoaffinity column	Sample extract: MeOH:H20 (80:20 *v*/*v*) and NaCl. After filtration, the extract was diluted in phosphate buffered saline (PBS), ad filtered again. IAC: The column was buffered with PBS and then the filtered sample was eluted through the column with ACN at a flow rate of 5 mL/min. The column was washed twice with distilled water and air-dried. After that, the eluate was dried and derivatized, and an aliquot was used for the HPLC analysis.	67	-	<LOD–91.7	Most of the analyzed samples exceeded the levels of AFB1 and AFs (2 and 4 μg/kg, respectively) tolerated in cereals in the European Community	[63]
	AFB2					<LOD–12.1		
	AFG1					<LOD–78.7		
	AFG2					<LOD–31.0		
	AFs					<LOD–138.6		
Rice	Total mycotoxins	QuEChERS	Extraction step: Solvent: ACN:HOAc (99:1 *v*/*v*) Salts: mixture of anhydrous MgSO_4,_ NaCl, (CH_2_COONa)_2_·2H_2_O and C_6_H_6_Na_2_O_7_·1.5H_2_O (4:1:1:0.5). After being vortexed and centrifuged, the supernatant was collected in a PTFE tube for the purification step, containing anhydrous magnesium sulfate and a C18 sorbent (This process is imperative toreduce the quantity of lipids and eliminate the excess of water, simplifying the evaporation). After centrifugation, the supernatant was evaporated and reconstituted in MeOH:H_2_O (70:30 *v*/*v*). After filtration, the extract was collected into a LC vial.	24	2013	ND	The target mycotoxins were not detected in any of the samples.	[4]
Rice	AFB1, AFB2, AFG1, AFG2, OTA, DON, ZEA, FB1, FB2, HT2, T2	d-SPE, QuEChERS	Extraction step: Solvent: water + 10% FA in ACN Salts: mixture of anhydrous MgSO_4_, NaCl, tri-Na and di-Na Purification step (d-SPE) ACN extract + MgSO4 + C18 + Al-N + PSA. After centrifugation, the extract was evaporated to dryness under a N_2_ steam, and reconstituted using mobile phase A:B (1:1 *v*/*v*). The samples were then filtered and collected in a vial for injection.	20	-	ZEA was detected in 2 rice samples and AFB1 was detected in 6 rice samples	The contamination levels were below the EU limits for typical foods and feeds.	[64]
Rice	AFB1	SPE	The samples were extracted with 20 mL ACN/water/glacial acetic acid (79:20:1, *v*/*v*/*v*. Aliquots of 500 μL extracts were transferred into glass vials containing an equal volume of ACN/water/acetic acid (20:79:1, *v*/*v*/*v*).	65	April 2010–April 2011	<LOQ–30.83	All the samples were contaminated with at least one mycotoxin. 3 rice samples exceeded the limit established in EU and Iran for AFB1 (5 μg/kg); ZEA was detected in 19 out of 65 samples in high levels.	[65]
	AFB2					0.6–1.26		
	FB1					54.48–176.58		
	OTA					0.65–11.54		
	ZEA					4.95–215.46		
Rice	T-2 toxin	SPE using multi - walled carbon nanotubes as sorbents	The samples were macerated using 10 mL of ACN/water (84:16, *v*/*v*) and then ultrasonicated. After centrifugation, the supernatant was collected and dried using nitrogen gas. The residues were reconstituted in ACN/water (20:80, *v*/*v*) and then diluted with water. This solution was passed through the multi-walled carbon nanotubes sorbents. The cartridges were eluted with MeOH containing 1% FA, and the eluate was evaporated using nitrogen gas. The residues were re-dissolved in ACN/water containing ammonium acetate (30:70, *v*/*v*), filtered and collected in a vial for injection.	10	-	6.13 (1/10 samples)	EFSA has established a TDI of 100 μg/kg body weight for the total of T-2 and HT-2 toxins	[66]
	HT-2 toxin					11.81 (1/10 samples)		
White rice	AFB1	SPE, Immunoaffinity columns	AFs: Solvent: ACN:water (90:10 *v*/*v*) After filtration, the supernatant was diluted with deionized water. IAC: the dilute filtrate was eluted at a flow rate of 3–4 drops/s using HPLC grade MeOH and washed with water. After evaporation under a nitrogen stream, a mixture of ACN:water (1:9 *v*/*v*) was added to the vials. OTA: Solvent: ACN:water (90:10 *v*/*v*) After filtration, the sample was mixed in PBS and filtered using a glass microfiber. After filtration, 10 mL of filtrate were mixed with acetic acid and passed through the IAC. IAC: The sample was eluted with MeOH and collected in a vial.	34	August 2012–March 2013	7.70 ± 0.89	25% of the samples of brown rice were above the maximum permitted level at EU for AFB1, and 32% for total AFs. 19% of the samples of rice and rice products were found positive and 14% were found above the EU maximum content for OTA (5 μg/kg)	[67]
	AFs					11.9 ± 1.20		
	OTA					8.50 ± 0.60		
Brown rice	AFB1			28		8.91 ± 1.20		
	AFs					12.4 ± 0.98		
	OTA					7.84 ± 0.90		
Rice flour	AFB1			30		3.51 ± 1.20		
	AFs					5.20 ± 0.82		
	OTA					4.91 ± 1.53		
Sweet puffed Rice balls	AFB1			22		2.90 ± 0.85		
	AFs					4.30 ± 1.25		
	OTA					3.87 ± 0.75		
Rice cookies	AFB1			28		3.18 ± 0.40		
	AFT					5.40 ± 0.92		
	OTA					3.18 ± 0.60		
Rice sweets	AFB1			21		4.10 ± 1.30		
	AFT					5.70 ± 0.80		
	OTA					5.10		
Rice noodles	AFB1			20		3.60 ± 0.85		
	AFT					3.60 ± 0.85		
	OTA					ND		
Rice bread	AFB1			25		2.40 ± 0.43		
	AFT					2.40 ± 0.43		
	OTA					ND		
Brown rice	AFT	QuEChERS	Extraction step: Solvent: water and HOAc in ACN (10% *v*/*v*) Salts: mixture of anhydrous MgSO_4,_ NaCl, (CH_2_COONa)_2_·2H_2_O and C_6_H_6_Na_2_O_7_·1.5H_2_O Centrifugation in order to separate the aqueous phase from the organic phase and then collection of the supernatant for the Purification step: C18 silica sorbent, anhydrous magnesium sulfate, PSA and silica. After centrifugation, the supernatant was collected into a vial. After evaporating the remaining ACN and reconstituting in water with a 1:1 (*v*/*v*) ratio of 0.1% (*v*/*v*) FA:MeOH, the sample was filtered and collected in the UHPLC-MS/MS vial	14	-	N.D	6 samples were contaminated with one or more mycotoxins. The levels determined were below the maximum limits of EU regulation.	[37]
	OTA					N.D		
	DON					N.D		
	FB1					2.49–5.41		
	FB2					4.33		
Infant cereals based on rice	AFB1	SPE	Solvent: ACN:water: FA (80:19.9:0.1 *v*/*v*/*v*) After centrifugation, the supernatant was transferred into an HPLC vial and a [^13^C] labelled working solution was added.	20	March 2012–June 2012	1/20 (5.9)	1 sample exceeded the EU limit for AFB1.	[68]
	AFB2					4/20 (1.1–5.0)		
	AFG1					ND		
	AFG2					ND		
	DON					7/20 (1.4–55.0)		
	HT-2 toxin					ND		
	T-2 toxin					3/20 (1.1–3.6)		
	FB1					ND		
	FB2					ND		
	OTA					2/20 (1.3–1.4)		
	ZEN					1/20 (9.0)		
Rice wine	OTA	VADLLME (Vortex-assisted dispersive liquid-liquid microextraction)	After centrifugation, the sample pH was adjusted to 4.0–4.3 using 4M NaOH or HCL solutions. Extraction solvent: dichloromethane Dispersive solvent: ACN The mixture was vortexed. After centrifugation, the sediment phase was evaporated to dryness using a nitrogen stream at 50 °C. The residues were reconstituted in a MeOH/water solution (50:50, *v*/*v*) and filtrated through a nylon filter membrane.	8	2016	0.20 μg/L (1/8 sample)	The contamination levels did not exceed the maximum residue limit set by EU (2 μg/L)	[69]
	AFs					ND		
Brown rice	AFB1	Immunoaffinity column	Sample extract: MeOH:Water (80:20, *v*/*v*) with NaCl. After filtration, the solution was diluted in phosphate buffered saline (PBS). IAC: The solution was applied to the IAC at a flow rate of 2–3 mL/min. The column was washed with distilled water, and the sample was eluted with MeOH and diluted with milli Q water	187	-	<LOD–0.069	Less than 14% of the rice samples were contaminated with aflatoxins, but two of the market samples were well above the maximum tolerable limit.	[70]
	AFB2					<LOD		
	AFG1					<LOD		
	AFG2					<LOD		
	AFs					<LOD–0.069		
Red rice	AFB1					<LOD–63.32		
	AFB2					<LOD–8.591		
	AFG1					<LOD		
	AFG2					<LOD		
	AFs					<LOD–70.91		
Rice	AFs	IAC	Sample extract: Sodium chloride and LC grade MeOH 70%. After filtration, the mixture was diluted in PBS and then filtered again. IAC: elution of the sample with 100% LC grade MeOH and LC grade water	100	2017	4,9 (1 sample)	The level is above the legislated levels.	[29]
	DON ZEA	Stable isotope dilution assay	Solvent: ACN:water:FA (80:19.9:0.1 *v*/*v*/*v*). After centrifugation, the supernatant was resuspended in a mobile phase composed by 70% of water:MeOH:acetic acid (94:5:1, *v*/*v*/*v*) and 30% of water:MeOH:acetic acid (2:97:1, *v*/*v*/*v*).			ND (0/100 samples) 15/100 samples (90,56–126,31)	ZEA levels were higher in 36% of the samples, than the current maximum limit established by Brazilian and European regulation	
Rice	AFB1	QuEChERS	Extraction step: Solvent: ACN Salts: mixture of MgSO_4_ and NaCl. Centrifugation in order to separate the aqueous phase from the organic phase and then collection of the top organic phase for the Purification step: C18 silica sorbent and magnesium sulfate After centrifugation, the supernatant was collected into a vial. After evaporating the remaining ACN and adding MeOH, the sample was filtered and collected in a new vial.	47	April 2013	Mean: 3.9 (<LOQ–14)	Most samples were contaminated with more than one mycotoxin (8 different mycotoxins were detected in 2 rice samples). Contamination levels higher than the EU limit for AFB1 were found in 42% of rice samples and for Aft in 32% of the same samples. OTA levels were also higher than the regulated from the EU.	[2]
	AFG1					Mean: 3.3 (<LOQ–17)		
	AFs					Mean: 5.8 (<LOQ–33)		
	OTA					Mean: 6.3 (<LOQ–15)		
	FB1+FB2					Mean: 6.0 (2.7–13)		
	ZEA					Mean: 6.6 (<LOQ–7.5)		
Ready to eat rice	DON	QuEChERS	Extraction step: Solvent: ACN Salts: mixture of MgSO_4_ and NaCl. Purification step: Anhydrous MgSO_4_ and a C18 silica sorbent. After centrifugation, the extract was filtered using a syringe nylon filter, into the LC-MS/MS vial; For GS-MS/MS the supernatant was evaporated to dryness using a nitrogen flow.	38	September 2016–December 2016	0.29	All levels were in accordance with the EU legislation	[71]
	HT-2 toxin					3.47		
	T-2 toxin					0.52		
	ZEA					0.13		
	AFG2					0.17		
Polished rice Unhusked rice	AFB1	QuEChERS	Extraction step: Solvent: ACN aqueous solution (95:5, *v:v*) Salts: anhydrous magnesium sulfate and sodium chloride. Purification step: After vortex and centrifugation, the supernatant was collected and filtered into the LC-MS/MS vial		78 22	2 samples (0.003–0.14) N.D.	The levels of AFB1 were lower than the regulation limit in EU (2 μg/kg)	[12]
Polished rice Unhusked rice	AFB1	QuEChERS	Extraction step: Solvent: ACN aqueous solution (95:5, *v:v*) Salts: anhydrous magnesium sulfate and sodium chloride. Purification step: After vortex and centrifugation, the supernatant was collected and filtered into the LC-MS/MS vial	78 22	-	2 samples (0.003–0.14) N.D.	The levels of AFB1 were lower than the regulation limit in EU (2 μg/kg)	[12]
Rice	AFB1	QuEChERS	Extraction step: Solvent: ACN containing 1% acetic acid Salts: mixture of anhydrous magnesium sulfate and sodium chloride. Purification step: Anhydrous magnesium sulfate and a C18 sorbent. After vortex and centrifugation, the supernatant was collected and filtered into the LC-MS/MS vial	144 (bulk sample > 0.5 kg)	October 2016–September 2017	13/144 samples (ND–93 μg/kg)	The levels of AFB1 were lower than the regulation limit in Vietname (5 μg/kg), but higher than the EU limits (2 μg/kg)	[27]
	FB1					3/144 samples (ND–675)		
	OTA					ND		
	ZEA					ND		

Legend: ACN—acetonitrile; AFB1—Aflatoxin B1; AFB2—Aflatoxin B2; AFG1—Aflatoxin G1; AFG2—Aflatoxin G2; AFs—Total aflatoxins; C8—octysilica; (CH_2_COONa)_2_ 2H_2_O—sodium citrate tribasic dihydrate; C_6_H_6_Na_2_O_7_·1.5H_2_O—sodium citrate dibasic sesquihydrate; DON—Deoxynivalenol; d-SPE—Dispersive Solid Phase Extraction; EFSA—European Food Safety Authority; EU—European Union; FA—Formic Acid; FB1—Fumonisin B1; FB2—Fumonisin B2; GC—Gas Chromatography; HCl—hydrogen chloride; HOAc—Acetic Acid; HPLC—High Performance Liquid Chromatography; IAC—Immunoaffinity Column; LC—Liquid Chromatography; LOD—Limit of Detection; MeOH—methanol; MgSO_4_—Magnesium Sulfate; MSPD—matrix solid phase dispersion; NaCl—Sodium Chloride; NaOH—Sodium hydroxide; ND—Not Detected; OTA—Ochratoxin A; PBS—phosphate buffered saline; PSA—Primary/Secondary amine; SPE—Solid Phase Extraction; TDI—Tolerable Daily Intake; UHPLC-MS/MS—Ultra High Performance Liquid Chromatography coupled with tandem mass spectrometry; ZEA—Zearalenone.

**Table 3:** Liquid chromatography analytical methodologies to determine mycotoxins in rice and rice products.

Mycotoxins Analyzed	Analytical Technique	Conditions	Analytical Column	LOD and LOQ (μg/kg)	Ref.
OTA	LC-FD	Mobile phase: MeOH- FA 0.1M (70:30 *v*/*v*) Flow rate: 0.7 mL/min λ_Excit max_: 333 nm and λ_Emis max_: 460 nm	C18 column (150 × 4.6 mm, 5 μm)	LOD: 0.05; LOQ: 0.19	[61]
AFT (AFB1, AFB2, AFG1 and AFG2)	LC - MS/MS	Mobile phase: A - MeOH; B - water with 0.1% acetic acid; Elution: Gradient; Column temperature: 30 °C; Injection volume: 10–0 μL; Flow: 0.25 mL/min; Electrospray ionization (ESI); Capillary potential: 3 kV; Nebulizing, desolvation and cone gas: nitrogen; Desolvation gas temperature: 400 °C Source temperature: 120 °C;	C18 column (2.1 × 50 mm, 1.9 μm)	LOD: 0.01–25; LOQ: 0.02–40	[62]
OTA					
ZEA					
DON					
FB1					
FB2					
T2 toxin					
HT-2 toxin					
Aft (AFB1, AFB2, AFG1 and AFG2)	Fluorescence detector	HPLC-FD Mobile phase: MeOH: Water [40:60 *v*/*v*] adjusted with 350 μl of 4 M nitric acid and 119 mg of potassium bromide per 1 L of mobile phase. Column temperature: 40 °C; Injection volume: 100 μL; Flow: 1 mL/min; λ_Excit max_ = 362 nm, and λ_Emis max_ = 426 nm (for AFB1 and AFB2) and λ_Emis max_ = 256 nm for AFG1 and AFG2)	Inertsil ODS-3V C18 column (4.6 × 150 mm, 5 μm)		[28]
Aft (AFB1, AFB2, AFG1 and AFG2)	Fluorescence detector	HPLC-FD: Mobile phase: Water:ACN:MeOH [65:15:20 *v*/*v*/*v*] degassed for 30 min using vacuum filtration Column temperature: 20 °C; Injection volume: 20 μL; Flow: 1.0 mL/min; λ_Excit max_ = 360 nm, and λ_Emis max_ = 450 nm	Reverse phase C18 column (4.6 mm × 250 mm, 5 μm)	LOD: 0.4–0.6; LOQ: 1.2–1.9	[63]
Total mycotoxins (AF, OTA, T-2 and HT-2 toxins, DON, ZEA, FB1)	LC-ESI-MS/MS	Mobile phase: H2O:MeOH 9:1 with 5 mM ammonium acetate; Elution: Gradient; Column temperature: 30 °C; Injection volume: 20 μL; Flow: 0.3 mL/min; Electrospray ionization (ESI); Ionization mode: Positive; Capillary potential: 2.9 kV; Nebulizing, desolvation and cone gas: nitrogen; Collision gas: argon Cone gas flow: 80 L/h Flow of desolvation gas: 650 L/h; Desolvation gas temperature: 350 °C; Source temperature: 140 °C;	Silica-based reversed-phase C18 Atlantis T3 (150 mm × 2.1 mm × 5 μm)	LOD: 0.11–59.9; LOQ: 0.37–199	[4]
AFB1, AFB2, AFG1, AFG2, OTA, DON, ZEA, FB1, FB2, HT2, T2	UHPLC-MS/MS (micromass quattro premier XE triple- quadrupole mass spectrometer)	Mobile phase: A - 0.5% FA in 5 mM aqueous ammonium formate; B - ACN:MeOH (1:1, *v*/*v*) Elution: Gradient; Column temperature: 40 °C; Injection volume: 5 μL; Flow: 0.25 mL/min; Electrospray ionization (ESI); Ionization mode: Positive (except for ZEA)	C18 column (1.7 μm, 100 × 2.1 mm), with a pre-column (1.7 μm, 5 × 2.1 mm)	LOD: 0.5–15; LOQ: 1.7–50	[64]
AFB1	HPLC - ESI - MS/ MS	Column temperature: 25 °C; Nebulizing, desolvation and cone gas: nitrogen; Source temperature: 550 °C	C18 column (5 μm, 30 × 2 mm)	LOD: 0.03–2.5; LOQ: 0.3	[65]
AFB2				LOD: 0.03–2.5; LOQ: 0.6	
FB1				LOD: 0.03–2.5; LOQ: 7	
OTA				LOD: 0.03–2.5 LOQ: 0,6	
ZEN				LOD: 0.03–2.5; LOQ: 2	
T-2 toxin	UHPLC-MS/MS	Mobile phase: A - Water with 5 mmol/L ammonium acetate; B - MeOH Elution: Gradient; Column temperature: 40 °C; Injection volume: 5 μL; Flow: 0.4 mL/min; Electrospray ionization (ESI); Ionization mode: Positive; Flow of desolvation gas: 1000 L/h; Flow of cone gas: 30 L/h Desolvation gas temperature: 500 °C Source temperature: 150 °C;	C18 column (100 × 3.0 mm, 2.7 μm)	LOD: 0.01; LOQ: 0.02	[66]
HT-2 toxin				LOD: 0.03; LOQ: 0.10	
Aflatoxins	HPLC-FD	Mobile phase: ACN:MeOH:water [20:20:60 *v*/*v*/*v*] Flow rate: 1 mL/min λ_Excit max_: 360 nm and λ_Emis max_: 440 nm	C18 (4.6 × 250 mm, 5 μm)	AFB1: LOD 0.04; LOQ 0.20; AFB2: LOD 0.10; LOQ 0.30; AFG1: 0.04; LOQ 0.20 AFG2 LOD 0.10; LOQ 0.30	[67]
OTA		Mobile phase: ACN:water:acetic acid [47:51:2 *v*/*v*/*v*] Flow rate: 1 mL/min λ_Excit max_ = 333 nm and λ_Emis max_ = 460 nm		LOD: 0.06; LOQ: 0.18	
Aft (AFB1, AFB2, AFG1, AFG2)	HPLC - ESI - MS/ MS	Mobile phase: A - 0.5% (*v*/*v*) FA in water containing 5 mM ammonium formate; B - MeOH Elution: Gradient; Column temperature: 40 °C; Injection volume: 10 μL; Flow: 0.3 mL/min; Electrospray ionization (ESI); Ionization mode: Negative and Positive Collision energy: 25 eV Cell accelerator voltage: 3V Capillary voltage: 3 kV; Nozzle voltage: 1000V Gas flow: 16 L/min; Gas temperature: 150 °C	C18 column (100 × 2.1 mm, 1.8 μm)	LOD: 0.27–0.39; LOQ: 0.82–1.2	[37]
OTA				LOD: 0.47; LOQ: 1.5	
DON				LOD: 5.0; LOQ: 15	
FB1, FB2				LOD: 0.48; LOQ: 1.5	
AFB1	HPLC - ESI - MS/ MS	Mobile phase: A - 0.1% FA in water; B - 0.1% FA in MeOH, both containing 5 mM ammonium formate; Elution: Gradient; Column temperature: 35 °C; Flow: 0.3 mL/min; Electrospray ionization (ESI); Ionization mode: Positive Flow of desolvation gas: 10 L/min; Desolvation gas temperature: 300 °C Nebulizer: 45 psi Sheath gas temperature: 350 °C Flow rate: 11 L/min Capillary voltage: 3500 V; nozzle voltage: 0 V	C18 column (100 × 2.1 mm, 1.8 μm)	LOD: 0.1; LOQ: 0.5	[68]
AFB2				LOD: 0.5; LOQ: 1.0	
AFG1				LOD: 0.1; LOQ: 0.5	
AFG2				LOD: 0.5; LOQ: 1.0	
DON				LOD:10.0; LOQ: 50.0	
HT-2 toxin				LOD: 1.0; LOQ: 5.0	
T-2 toxin				LOD: 0.05; LOQ: 0.1	
FB1				LOD: 5.0; LOQ: 10.0	
FB2				LOD: 1.0; LOQ: 5.0	
OTA				LOD: 0.1; LOQ: 0.5	
ZEA				N.D.	
AFB1	HPLC - MS/MS	Mobile phase: A - MeOH; B - water with 0.1% FA Elution: Gradient; Column temperature: 40 °C; Injection volume: 5 μL; Flow: 0.3 mL/min; Electrospray ionization (ESI); Ionization mode: Positive Capillary potential: 4.0 kV; Vaporizer temperature: 300 °C Capillary temperature: 350 °C	C18 column (100 × 3.0 mm, 2.7 μm)	LOD: 0.05; LOQ: 0.1	[69]
AFB2				LOD: 0.05; LOQ: 0.1	
AFG1				LOD: 0.1; LOQ: 0.2	
AFG2				LOD: 0.05; LOQ: 0.1	
OTA				LOD: 0.05; LOQ: 0.1	
AFB1	HPLC-FD	Mobile phase: water:ACN:MeOH (6:2:3, *v*/*v*/*v*), containing KBr and nitric acid Elution: Gradient; Injection volume: 20 μL; Flow: 1 mL/min; λ_Excit max_ = 362 nm and λ_Emis max_ = 455 nm (for AFG1 and AFG2) and 425 (for AFB1 and AFB2)	C18 column (4.6 × 150 mm, 5 μm)	LOD: 0.016; LOQ: 0.054	[70]
AFB2				LOD: 0.012; LOQ: 0.039	
AFG1				LOD: 0.011; LOQ: 0.038	
AFG2				LOD: 0.004; LOQ: 0.012	
DON	LC-MS/MS	Mobile phase: water:MeOH:ACN (600:200:200, *v*/*v*/*v*) was added to119 mg potassium bromide and 47.6 μL nitric acid Elution: Gradient; Flow: 1 mL/min; Electrospray ionization (ESI); Ionization mode: Positive Capillary temperature: 208 °C; Vaporizer temperature: 338 °C; Spray voltage: 4500 V; Sheath gas pressure: 60 bar	RP - C18 column (4.6 × 150 mm, 5 μm)	LOD: 0.005; LOQ:0.025	[29]
ZEA				LOD: 0.01; LOQ: 0.025	
AFB1	UHPLC-MS/MS	Mobile phase: A – 0.1% FA in water; B - MEOH:ACN (1:1 *v*/*v*) Elution: Gradient; Column temperature: 40 °C; Injection volume: 1 μL; Flow: 0.4 mL/min; Electrospray ionization (ESI); Ionization mode: Positive and negative Capillary potential: 1.5 kV; Flow of desolvation gas: 1000 L/h; Desolvation gas temperature: 500 °C; Source temperature: 150 °C;	C18 column (1.6 μm, 2.1 × 100 mm)	LOD: 0.05; LOQ: 0.25	[2]
AFG1				LOD: 0.12 LOQ: 0.25	
Aft (AFB1, AFB2, AFG1 and AFG2)				-	
OTA				LOD: 0.25; LOQ: 0.62	
FB1 + FB2				LOD: 0.5; LOQ: 1	
ZEA				LOD: 2.5; LOQ: 5	
DON	LC-MS/MS	Mobile phase: A - MeOH (5mM ammonium formate and 0.1% FA); B - water (5mM ammonium formate 0.1% FA; Elution: Gradient; Column temperature: 25 °C; Injection volume: 20 μL; Flow: 0.25 mL/min;	Reverse analytical column C18 (3 μm, 150 × 2 mm ID) and a guard column C18 (4 × 2 mm ID, 3 μm)	LOD: 0.04–1.5; LOQ: 0.13–5	[80]
HT-2 toxin					
T-2 toxin					
ZEA					
AFG2					
AFB1	LC- MS/MS	Mobile phase: A - aqueous FA solution with ammonium formate; B - ACN Elution: Gradient; Injection volume: 5 μL; Ionization: electrospray ionization (ESI)	ShimadzuShim-pack XR-ODS III column	LOD: 0.03 LOQ: 0.5	[12]
AFB1	LC - MS/MS	Mobile phase: A - MeOH; B- ammonium acetate 10 mM Elution: Gradient; ESI mode: positive (for AFB1 and FB1) and negative (for OTA and ZEA) Ionization: electrospray ionization (ESI)	C18 column (4.6 × 150 mm, 2.7 μm)	LOD: 0.1 LOQ: 0.3	[12]
FB1					
OTA					
ZEA					
FB1	RP-HPLC/ESI-TOFMS	Mobile phase: A – water containing 0.1% (*v*/*v*) formic acid; B - MeCN containing 0.1% (*v*/*v*) formic acid Flow: 0.2 mL min^−1^ Column Temperature: 40 °C Elution: Gradient Injection Volume: 1 μL ESI mode: positive ESI parameters: drying gas (N_2_) flow and temperature, 10.0 L min^−1^ and 350 °C; nebulizer gas (N_2_) pressure, 20 psi; capillary voltage, 3500 V; TOFMS parameters: fragmentor voltage, 170 V; skimmer potential: 70 V; OCT 1 RF Vpp: 250 V	ODS H80 (250 mm × 2.1 mm, 4 μm)	-	[81]
FB1	RP-HPLC/ESIITMS	Mobile phase: A – water containing 0.1% (*v*/*v*) formic acid; B - MeCN containing 0.1% (*v*/*v*) formic acid Flow: 0.2 mL min^−1^ Column Temperature: 40 °C Elution: Gradient Injection Volume: 1 μL ESI mode: positive ESI parameters: spray chamber temperature, 55 °C; drying gas (N2) pressure and temperature, 20 psi and 350 °C, respectively; nebulizer gas (N2) pressure, 60 psi; needle voltage, 4000 V; spray shield voltage, 600 V; general parameters: maximum scan times, 2.71; mscans averaged, 3; data rate, 0.37 Hz; multipier offset, 0; Ionization control parameters: target TIC, 100%; maximum ion time, 500,000 ms MS2 parameters: capillary voltage, 139 V; RF loading, 75%; isolation window, 3 *m*/*z*; high mass ejection factor, 100%; waveform type, resonant; excitation storage level, 196.4 *m*/*z*; excitation amplitude, 2.83 V; excitation time, 10 ms; RF, modulate; number of frequencies, 1.	ODS H80 (250 mm × 2.1 mm, 4 μm)	-	[81]
AFB1	UHPLC/TOFMS	Mobile phase: A - water/ methanol/acetic acid 94:5:1 (*v*/*v*/*v*); B - methanol/water/acetic acid 97:2:1 (*v*/*v*/*v*) Flow rate: 0.2 mL·min^−1^ ESI mode: positive MS parameters: capillary voltage 6000 V, nebuliser pressure 2 bars, dry gas temperature 200 °C and dry gas flow 7 L min^−1^	C18 column (1.8 µm, 2.1 × 100 mm)	LOD:1 LOQ: 2	[82]
AFB2				LOD: 2 LOQ: 3	
AFB2				LOD: 1 LOQ:1	
AFG2				LOD: 1 LOQ: 3	
OTA				LOD: 9 LOQ: 18	
DON				LOD: 24 LOQ: 48	
FB1				LOD: 16 LOQ: 32	
HT — 2 Toxin				LOD: 20 LOQ: 41	
T-2 Toxin				LOD: 2 LOQ: 5	
ZEA				LOD: 39 LOQ: 77	
AFB1	UHPLC/TOFMS	Mobile phase: A - water/ methanol/acetic acid 94:5:1 (*v*/*v*/*v*); B - methanol/water/acetic acid 97:2:1 (*v*/*v*/*v*) Flow rate: 0.2 mL·min^−1^ ESI mode: positive MS parameters: capillary voltage 6000 V, nebuliser pressure 2 bars, dry gas temperature 200 °C and dry gas flow 7 L min^−1^	C18 column (1.8 µm, 2.1 × 100 mm)	LOD: 4 LOQ: 8	[82]
AFB2				LOD: 4 LOQ: 9	
AFG1				LOD: 7 LOQ: 14	
AFG2				LOD: 3 LOQ: 5	
OTA				LOD: 8 LOQ: 17	
DON				LOD: 29 LOQ: 59	
FB1				LOD: 10 LOQ: 19	
HT -2 Toxin				LOD: 7 LOQ: 15	
T-2 Toxin				LOD: 6 LOQ: 11	
ZEA				LOD: 22 LOQ: 45	

Legend: ACN—acetonitrile; AFB1—Aflatoxin B1; AFB2—Aflatoxin B2; AFG1—Aflatoxin G1; AFG2—Aflatoxin G2; AFs—Total aflatoxins; DON—Deoxynivalenol; ESI—Electrospray Ionization; FA—Formic Acid; FB1—Fumonisin B1; FB2—Fumonisin B2; FD—Fluorescent Detector; H_2_O—Water; HPLC—High Performance Liquid Chromatography; LC—Liquid Chromatography; LOD—Limit of Detection; LOQ—Limit of Quantification; MeOH—Methanol; MS/MS—Tandem mass spectrometry; OTA —Ochratoxin A; RP—Reverse Phase; UHPLC—Ultra High Performance Liquid Chromatography; ZEA—Zea; RP-HPLC/ESI-TOFMS—Reversed-phase High Performance Liquid Chromatography/Electrospray Ionization Time-of-Flight Mass Spectrometry; RP-HPLC/ESIITMS—Reversed-Phase High Performance Liquid Chromatography/Electrospray Ionization Ion Trap Mass Spectrometry; UHPLC/TOFMS—Ultra High Performance Liquid Chromatography/Time-of-Flight Mass Spectrometry.

**Table 4:** Comparison of MS/MS systems (TQ MS, Q-TOF MS and Orbitrap MS), adapted from [88,89].

	Strengths	Limitations
**TQ MS**	Highest sensitivity (MRM) Wide dynamic range of detection Lower cost	Low mass resolution
**Q-TOF MS**	High mass resolution Wide mass range Medium dynamic range of detection High sensitivity	Low sensitivity than TQ MS MRM mode
**Orbitrap MS**	High mass resolving power (up to 200,000) Increased space- charge capacity at higher masses due to the independence of trapping potential and larger trapping volume (in contrast to FTICR and quadrupole traps) High mass accuracy (1–2 ppm) High dynamic range (around 5000)	Expensive

Legend: TQ-MS- Triple quadrupole MS; Q-TOF MS- Quadrupole Time-of-Flight MS.

**Table 5:** RASFF notifications due to mycotoxins contamination from 2019 to 06/07/2022.

Date	Country	Origin Country	Product	Mycotoxin	Levels (µg/kg)
22/02/2019	Italy	Pakistan	Basmati rice	AFB1	4.3
22/02/2019	Belgium	Italy	Organic brown rice	OTA	14.1
01/03/2019	Belgium	Pakistan	Basmati rice	AFB1	6.8
01/03/2019	Italy	Pakistan	Basmati rice	AFB1	19.9
				AFs	21.6
22/03/2019	Austria	Germany	Organic brown rice	AFB1	7.1
22/05/2019	France	Italy	Basmati rice	AFB1	4.49
02/08/2019	Germany	Netherlands	Basmati rice	AFB1	3.60
05/09/2019	Poland	Myanmar	Parboiled brown rice	AFB1	4.09
24/10/2019	Portugal	Myanmar	Rice	AFB1	19
28/11/2019	Switzerland	Sri Lanka	Roasted red rice flour	AFB1	15.6
				AFs	19
18/12/2019	Switzerland	Sri Lanka	Roasted red rice flour	AFB1	6.8
				AFs	8.2
27/02/2020	Switzerland	Sri Lanka	Parboiled rice	AFB1	3.4
15/06/2020	Sweden	Cambodia	Organic brown rice	AFB1	20.6
03/07/2020	Greece	Pakistan	Basmati rice	AFB1	5.6
				AFs	5.6
07/07/2020	Greece	Pakistan	Basmati rice	AFB1	6.3
				AFs	6.3
07/07/2020	Greece	Pakistan	Basmati rice	AFB1	6.0
				AFs	6.0
31/07/2020	Poland	Pakistan	Long grain brown rice	AFB1	6.54
				AFs	6.54
21/08/2020	Greece	Pakistan	Basmati rice	AFB1	4.6
				AFs	4.6
21/08/2020	Switzerland	United Kingdom	Basmati rice	OTA	8.3
01/09/2020	Switzerland	Sri Lanka	Red rice	AFB1	8.9
				AFs	11
				OTA	10.3
15/10/2020	Germany	India	Basmati rice	OTA	6.23
20/10/2020	Germany	Pakistan	Organic brown basmati rice	AFB1	14.3
				AFs	15.4
02/12/2020	Netherlands	India	Brown basmati rice	AFB1	24
				AFs	27
05/01/2021	Spain	Pakistan	White rice	AFB1	2.2–3.1
21/01/2021	Spain	Pakistan	White rice	AFB1	3
22/01/2021	Greece	Pakistan	Basmati rice	AFB1	3.1
28/01/2021	Netherlands	Pakistan	Organic brown basmati rice	OTA	11.2
04/03/2021	Netherlands	Pakistan	Organic brown basmati rice	AFB1	9.1
17/03/2021	Germany	Netherlands	Basmati rice	OTA	5.26
27/04/2021	Germany	Netherlands	Rice flour	AFB1	5.7 ± 2.5
27/05/2021	Germany	India	Basmati rice	OTA	4.94 ± 0.41
06/08/2021	Netherlands	Pakistan	Brown rice	AFB1	44
				AFs	49
10/08/2021	Belgium	Pakistan	Broken rice	AFB1	8.6
27/08/2021	Belgium	Pakistan	White broken rice	AFB1	8.6
14/12/2021	Switzerland	Sri Lanka	Rice	AFB1	6.3 ± 1.07
				AFs	6.59 ± 1.32
16/12/2021	Germany	Pakistan	Basmati Rice	AFB1	3.96 ± 1.60
06/01/2022	Belgium	Pakistan	Rice bran	AFB1	4.15
07/02/2022	Netherlands	Pakistan	Basmati Rice	AFB1	13
				AFs	15
14/02/2022	Netherlands	Pakistan	Golden sun basmati rice	AFB1	5
17/02/2022	Netherlands	Pakistan	Rice	AFB1	4.2
17/02/2022	Netherlands	Pakistan	Rice	AFB1	7
22/02/2022	Netherlands	Pakistan	Rice	AFB1	7
22/02/2022	Netherlands	Pakistan	Basmati rice	OTA	12
23/02/2022	Netherlands	India	Basmati rice	AFB1	4.2
23/02/2022	Netherlands	India	Basmati rice	OTA	6.8
				AFB1	3.1
25/02/2022	Netherlands	India	Basmati rice	AFB1	3.2
25/02/2022	Netherlands	India	Basmati rice	AFB1	3.4
28/02/2022	Belgium	Pakistan	Rice	AFB1	5.3
				AFs	6.5
02/03/2022	Netherlands	Pakistan	Rice	AFB1	7.3
10/03/2022	Netherlands	Pakistan	Super basmati brown rice (husked rice)	AFB1	11
				AFs	11
10/03/2022	Netherlands	Pakistan	Rice	AFB1	9.7
				AFs	9.7
11/03/2022	Netherlands	Pakistan	Super basmati brown rice (husked rice)	AFB1	4.7
11/03/2022	Netherlands	Pakistan	Super basmati brown rice (husked rice)	AFB1	14
				AFs	14
14/03/2022	Italy	India	Basmati rice	AFs	4.9 ± 2.0
14/03/2022	Netherlands	Pakistan	Super kernel basmati brown rice	AFB1	5.6
15/03/2022	Italy	Pakistan	Rice	AFB1	4.6 ± 2.0
15/03/2022	Italy	Pakistan	Rice	AFB1	7.2 ± 3.2 *
				AFS	7.9 ± 3.2 *
24/03/2022	Greece	Pakistan	Rice	AFB1	10.7 ± 2.1
				AFs	10.7 ± 2.1
29/03/2022	Netherlands	Pakistan	Rice	AFB1	10
				AFs	10
29/03/2022	Cyprus	India	Basmati rice	AFB1	5.82
31/03/2022	Netherlands	Pakistan	Rice	AFB1	12
				AFs	13
07/04/2022	Netherlands	Pakistan	Rice	AFB1	24
				AFs	26
07/04/2022	Netherlands	Pakistan	Rice	AFB1	15
				AFs	16
07/04/2022	Netherlands	Pakistan	Rice	AFB1	19
				AFs	20
13/04/2022	Netherlands	Pakistan	Super basmati brown rice (husked rice)	AFB1	18
				AFs	20
15/04/2022	Netherlands	Pakistan	Super kernel basmati brown rice	AFB1	8
15/04/2022	Netherlands	Pakistan	Super basmati brown rice	AFB1	5.1
19/04/2022	Netherlands	Pakistan	Rice	AFB1	11
27/04/2022	Netherlands	Pakistan	Super basmati brown rice	AFB1	9.1
				AFs	9.1
03/05/2022	Netherlands	Pakistan	Super basmati brown rice	AFB1	6.8
03/05/2022	Netherlands	Pakistan	Super kernel basmati brown rice	AFB1	7.2
04/05/2022	Netherlands	Pakistan	Super basmati brown rice	AFB1	8.5
				AFs	8.5
12/05/2022	Netherlands	Pakistan	Basmati brown rice (husked rice)	AFB1	11
				AFs	11
12/05/2022	Netherlands	Pakistan	Super basmati brown rice (husked rice)	AFB1	5.1
12/05/2022	Netherlands	Pakistan	Super basmati brown rice (husked rice)	AFB1	4.7
12/05/2022	Netherlands	Pakistan	Super basmati brown rice (husked rice)	AFB1	48
				AFs	53
12/05/2022	Ireland	India	Basmati rice	OTA	6.3 ± 0.2
18/05/2022	Netherlands	Pakistan	Rice	AFB1	23
				AFs	25
20/05/2022	Netherlands	Pakistan	Husked brown rice	AFB1	8.2
				AFs	8.2
20/05/2022	Cyprus	India	Basmati rice	OTA	16.5
27/05/2022	Netherlands	Pakistan	Super basmati brown rice	AFB1	7.1
01/06/2022	Spain	Pakistan	Basmati rice	AFB1	5.6 ± 24.2%
				AFs	5.6 ± 24.2%
20/06/2022	Slovenia	Pakistan	Basmati brown rice	AFB1	13.2 ± 2
				AFs	14 ± 2
30/06/2022	Netherlands	Pakistan	Rice	AFB1	7.1
01/07/2022	Netherlands	Pakistan	Rice	AFB1	4.7
04/07/2022	Netherlands	India	Rice	OTA	6.4
06/07/2022	Netherlands	India	Rice	OTA	9.2

Legend: Notifications of mycotoxins contamination in rice and rice products from 2019 to 2021; Adapted from RASFF portal. * mg/kg.

**Table 6:** Different decontamination means of mycotoxins in food, their advantages, and disadvantages, adapted from [98].

	Physical Decontamination	Chemical Decontamination	Biological Decontamination
Examples	Sorting Sieve cleaning Density segregation Washing De-hulling Steeping Extrusion cooking Steam heating Infrared heating Microwave heating Radio frequency heating Irradiation Cold plasma Photocatalytic detoxification	Organic acids Hydrochloric acid Ammonium hydroxide Hydrogen peroxide Sodium bisulphite Chlorinating agents Ozone Formaldehyde Natural substances such as herbs, spices, and their extracts	Bacteria Yeasts Mold Algae
Advantages	Effective against some mycotoxins Low change in food properties Does not involve usage of chemicals	Effective against some mycotoxins Affordable	Effective against some mycotoxins Inexpensive Environment friendly Does not involve usage of chemicals
Disadvantages	Impractical Might be limited to large-scale industries with sophisticated equipment Time-consuming Expensive In case of thermal treatment possible changes in color and food quality	Possible health effects Formation of toxic byproducts Enhancing bioavailability of masked mycotoxins Time consuming Environmentally toxic	Time consuming Impractical More effective in controlled laboratory settings

## Data Availability

Not applicable.
